# Peritoneal tuberculosis mimicking malignancy on FDG PET/CT in a patient with ankylosing spondylitis on adalimumab: a diagnostic challenge

**DOI:** 10.22038/aojnmb.2025.88636.1639

**Published:** 2026

**Authors:** Harish Goyal, Srinivas Ananth Kumar, Anirudh Abu Srinivasan, Manikya Y S, Dhritiman Chakraborty

**Affiliations:** 1 Department of Nuclear Medicine, Jawaharlal Institute of Postgraduate Medical Education Research, Puducherry, India; 2 Department of Nuclear Medicine, Institute of Post Graduate Medical Education and Research, Kolkata, West Bengal, India

**Keywords:** Extrapulmonary tuberculosis 18F-FDG PET/CT, Ankylosing spondylitis Adalimumab, Peritoneal tuberculosis Empirical therapy A B S T R A C T

## Abstract

Tumor necrosis factor-alpha (TNF-α) inhibitors, such as adalimumab, are integral in the managing refractory ankylosing spondylitis (AS). However, their immunosuppressive effects elevate the risk of reactivation of latent tuberculosis (TB), especially extrapulmonary TB (EPTB), which can present with non-specific symptoms and mimic malignancy.

We report the case of a 40-year-old man with ankylosing spondylitis who had been on long-term adalimumab therapy. He presented with a low-grade fever and weight loss. An ^18^F-FDG PET/CT scan revealed intense FDG uptake in the peritoneum, omentum, and mesentery. There was no ascites or visceral involvement; however, a few FDG-avid retroperitoneal, mediastinal, and right cervical lymph nodes were noted, along with a right pleural effusion and no lesions in the lung parenchyma. Despite a negative microbiological workup, empirical anti-tubercular therapy (ATT) was initiated due to strong clinical and imaging suspicion of tuberculosis. The patient showed significant clinical improvement, and a follow-up PET/CT scan six months later indicated complete metabolic resolution of the lesions.

This case underscores the diagnostic challenge of peritoneal TB in immunosuppressed individuals. It highlights the supportive role of FDG PET/CT in guiding empirical therapy and monitoring treatment response without microbiological confirmation.

## Introduction

 Ankylosing spondylitis (AS) is a chronic inflammatory spondyloarthropathy predominantly affecting the axial skeleton. Tumor necrosis factor-alpha (TNF-α) inhibitors, such as adalimumab, have become cornerstone therapies in refractory cases by effectively reducing inflammation and improving patient mobility (1). However, TNF-α blockade substantially increases the risk of tuberculosis (TB), particularly extrapulmonary TB (EPTB), with incidence rates reported to be up to 25 times higher than in the general population(2, 3). 

 EPTB constitutes approximately 20% of TB cases in immunocompetent individuals but occurs more frequently and with atypical presentations in immunosuppressed patients (4). Among these, peritoneal TB is particularly challenging due to its non-specific clinical features and radiological resemblance to peritoneal carcinomatosis or mesothelioma. Microbiological confirmation is often elusive, with diagnostic yields reported negative in up to 40% of cases (5).

 In this context, ^18^F-fluorodeoxyglucose positron emission tomography/computed tomography (^18^F-FDG PET/CT) has emerged as a valuable diagnostic tool. Its whole-body metabolic imaging can detect occult inflammatory lesions, identify various sites of involvement, and provide a means of non-invasive therapeutic monitoring. However, FDG uptake is non-specific, and PET/CT cannot reliably differentiate tuberculosis from malignancy, particularly in granulomatous infections. This is particularly relevant in immunocompromised patients with fever of unknown origin or unexplained systemic inflammation (6-8).

 We present a diagnostically challenging case of peritoneal TB in a patient on long-term adalimumab therapy. The patient exhibited metabolically active peritoneal thickening with a few FDG-avid lymph nodes on PET/CT, but no pulmonary or visceral organ involvement. Despite persistently negative microbiological findings, empirical anti-tubercular therapy was initiated based on clinical and imaging features. This case highlights the diagnostic complexity of EPTB in biologically immunosuppressed patients and underscores the supportive role of ^18^F-FDG PET/CT in guiding therapy and assessing response.

## Case report

 A 40-year-old male with a six-year history of ankylosing spondylitis (AS) had been receiving adalimumab (40 mg subcutaneously every two weeks) for more than two years, with satisfactory disease control. The patient had not received prophylactic anti-tuberculosis therapy, and screening for latent TB infection was performed before the initiation of adalimumab, which was negative. He presented with a two-month history of intermittent low-grade fever (37.5–38 °C) and unintentional weight loss of approximately 6 kg. He also reported mild breathlessness but denied gastrointestinal or urinary complaints.

 On physical examination, breath sounds were diminished over the right hemithorax; the remaining of the systemic examination was unremarkable. High-resolution computed tomography (HRCT) of the chest revealed a right-sided pleural effusion without associated pulmonary parenchymal abnormalities. Pleural fluid cytology was negative for malignant cells.

Laboratory investigations showed elevated inflammatory markers, with an erythrocyte sedimentation rate (ESR) of 38 mm/hr and a C-reactive protein (CRP) level of 14 mg/L. Routine hematological and biochemical profiles were within normal limits. Serological testing for HIV, hepatitis B, and hepatitis C was negative.

**Figure 1:**
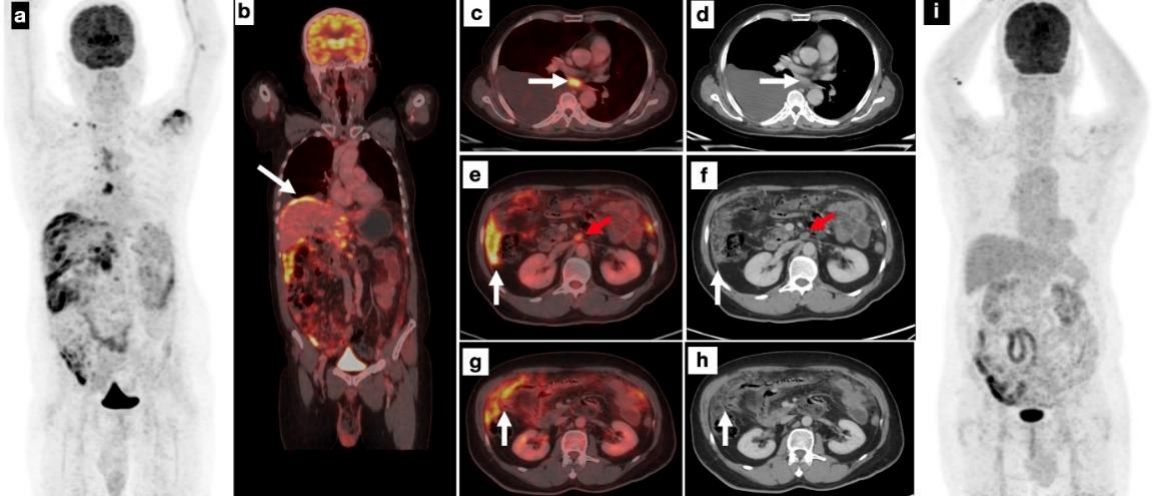
Maximum intensity projection (MIP) image of the baseline ^18^F-FDG PET/CT (**a**) demonstrates multiple hypermetabolic foci in the right lower cervical, mediastinal, and abdominal regions. Coronal fused PET/CT images (**b**) reveal increased FDG uptake along the peritoneal lining, particularly over the liver capsule. Axial fused PET/CT and corresponding CT images show right-sided pleural effusion and FDG-avid subcarinal lymph node (**c**, **d**), retroperitoneal lymphadenopathy (**e**, **f**, **red arrow**), and intense FDG uptake along the omentum and peritoneum (**e**, **g**, **white arrow**). Physiological FDG uptake is noted in the ascending colon, and focal uptake in the left shoulder region indicates bursitis (**a**). Follow-up ^18^F-FDG PET/CT after empirical anti-tubercular therapy shows complete metabolic resolution of previously observed lesions (**i**), confirming a favorable treatment response

 Given the non-specific systemic symptoms and suspicion of occult infection or malignancy, a whole-body ^18^F-FDG PET/CT was performed. Imaging demonstrated intense linear and nodular FDG uptake (SUV_max_=8.2) along the parietal and visceral peritoneum, omentum, and mesentery, without associated ascites or organ-based abdominal lesions. Additionally, FDG-avid lymphadenopathy was noted in retroperitoneal, mediastinal, and right cervical regions (including a subcarinal node with SUV_max_=4.2), along with a right pleural effusion (Figure 1a-h). Pulmonary parenchyma appeared normal. The leading imaging differentials included peritoneal tuberculosis (suggestive features), peritoneal carcinomatosis, and mesothelioma.

 Diagnostic peritoneal aspiration yielded scant fluid. Microbiological tests, including GeneXpert MTB/RIF assay, Ziehl–Neelsen stain for acid-fast bacilli (AFB), and mycobacterial culture, were all negative. In view of the clinical context, imaging features, and the high endemic burden of TB, first-line empirical anti-tubercular therapy (ATT), comprising isoniazid, rifampicin, pyrazinamide, ethambutol, and streptomycin, was initiated in line with local treatment protocols.

 The patient exhibited marked clinical improvement within six weeks, including fever and weight gain resolution. Inflammatory markers normalized (ESR decreased to 12 mm/hr; CRP to 5 mg/L). A follow-up ^18^F-FDG PET/CT scan performed at six months demonstrated complete metabolic resolution of the previously hypermetabolic peritoneal and nodal lesions (Figure 1-i), confirming both the diagnosis of peritoneal TB and the therapeutic response.

## Discussion

 This case illustrates the diagnostic challenges associated with peritoneal tuberculosis, particularly in patients receiving tumor necrosis factor-alpha (TNF-α) inhibitor therapy. While biologics such as adalimumab are highly effective in controlling disease activity in ankylosing spondylitis, their immuno-suppressive effects impair host defence mechanisms, significantly increasing the risk of reactivation or new infection with Mycobacterium tuberculosis, especially in extra pulmonary and disseminated forms (2, 3, 11, 12).

 Peritoneal TB remains a rare manifestation of EPTB, accounting for less than 1% of all TB cases (4). Its clinical presentation frequently overlaps with that of intra-abdominal malignancies, such as peritoneal carcinomatosis or mesothelioma, leading to considerable diagnostic uncertainty. As in our patient, the absence of ascites further complicates the differentiation. Additionally, the paucibacillary nature of the disease often limits microbiological confirmation, and mycobacterial cultures may take weeks to yield results, if positive at all (5). 

 In this diagnostic setting, ^18^F-FDG PET/CT is a valuable adjunct rather than a definitive tool. TB lesions often demonstrate intense FDG uptake due to underlying granulomatous inflammation. However, similar metabolic activity can also be seen in malignancy, necessitating careful interpretation in the appropriate clinical context (6, 7, 13). 

 Nevertheless, ^18^F-FDG PET/CT offers significant value by identifying occult disease, guiding biopsy decisions, and supporting early empirical therapy when integrated with clinical and epidemiological factors. It also provides a robust, non-invasive means of monitoring therapeutic response over time (8, 9).

 Although empirical ATT is sometimes debated in the absence of microbiological proof, current WHO and IDSA guidelines support its use in high TB burden settings when there is compelling clinicoradiological evidence (10). In this case, timely initiation of empirical ATT resulted in prompt clinical improvement and complete metabolic resolution on follow-up PET/CT, reinforcing the appropriateness of this strategy.

 Only a limited number of cases of peritoneal tuberculosis mimicking malignancy on FDG PET/CT have been reported (14, 15). This emphasizes the uniqueness of our case, where serial PET/CT imaging, rather than relying solely on initial baseline imaging, retro-spectively confirmed the diagnosis.

 In summary, peritoneal TB should remain a key differential diagnosis in patients presenting with peritoneal thickening and FDG-avid lesions, particularly those on TNF-α inhibitors. 

 The final diagnosis of peritoneal TB was based on the clinical context, high endemicity, empirical treatment, and the follow-up PET/CT showing complete metabolic resolution. This highlights the role of serial PET/CT imaging in supporting the diagnosis retrospectively, rather than as a definitive diagnostic tool. In regions where tuberculosis is common, ^18^F-FDG PET/CT can be beneficial and should be considered for diagnosing and monitoring culture-negative EPTB. This approach may improve outcomes by enabling timely treatment in these challenging diagnostic situations.
